# The membrane proteome of the mouse lens fiber cell

**Published:** 2009-11-24

**Authors:** Steven Bassnett, Phillip A. Wilmarth, Larry L. David

**Affiliations:** 1 Ophthalmology & Visual Sciences, Washington University School of Medicine, St. Louis, MO; 2 Biochemistry & Molecular Biology, Oregon Health & Science University, Portland, OR

## Abstract

**Purpose:**

Fiber cells of the ocular lens are bounded by a highly specialized plasma membrane. Despite the pivotal role that membrane proteins play in the physiology and pathophysiology of the lens, our knowledge of the structure and composition of the fiber cell plasma membrane remains fragmentary. In the current study, we utilized mass spectrometry-based shotgun proteomics to provide a comprehensive survey of the mouse lens fiber cell membrane proteome.

**Methods:**

Membranes were purified from young mouse lenses and subjected to MudPIT (Multidimensional protein identification technology) analysis. The resulting proteomic data were analyzed further by reference to publically available microarray databases.

**Results:**

More than 200 membrane proteins were identified by MudPIT, including Type I, Type II, Type III (multi-pass), lipid-anchored, and GPI-anchored membrane proteins, in addition to membrane-associated cytoskeletal elements and extracellular matrix components. The membrane proteins of highest apparent abundance included Mip, Lim2, and the lens-specific connexin proteins Gja3, Gja8, and Gje1. Significantly, many proteins previously unsuspected in the lens were also detected, including proteins with roles in cell adhesion, solute transport, and cell signaling.

**Conclusions:**

The MudPIT technique constitutes a powerful technique for the analysis of the lens membrane proteome and provides valuable insights into the composition of the lens fiber cell unit membrane.

## Introduction

Lens fiber cells are bounded by a plasma membrane no less remarkable in composition and organization than the cytoplasm it encapsulates. In fiber cells, the plasma membrane serves the same basic function as in any eukaryotic cell; namely, providing a semi-permeable barrier to segregate the contents of the cell from the exterior. Not surprisingly, therefore, many widely-expressed membrane proteins are found in lens membranes. Na,K-ATPase, for example, a ubiquitous integral membrane protein, is expressed strongly by lens fiber cells [1]. However, certain membrane proteins are unique to fiber cells, and their presence presumably reflects the singular role of the lens in the optical train of the eye.

Lens membrane proteins play central roles in the physiology and pathophysiology of the tissue. Genetic linkage studies have revealed that mutations in genes for Mip, Lim2, and other integral lens membrane proteins underlie inherited cataracts [2] and polymorphisms in the membrane receptor EphA2, may predispose to the much more common, age-related cataracts [3]. Fiber cell membrane proteins also contribute directly to the optical quality of the lens. Thus, targeted disruption of the Lim2 gene in the mouse lens causes profound disturbances in the internal refractive properties of the tissue, leading to a lens that no longer focuses sharply [4].

Despite the pivotal role of lens membrane proteins, our understanding of the composition and organization of the fiber cell plasma membrane remains fragmentary. The systematic analysis of membrane protein expression has been problematic due to the hydrophobic nature of membrane proteins and associated difficulties in solubilizing and resolving them on 2D gels. Shotgun methods, in which samples are treated with proteases and the resulting mixture of peptides are identified by mass spectrometry, offer a viable alternative. In particular, the development of MudPIT (Multidimensional protein identification technology) applications has facilitated the analysis of complex membrane proteomes [5-7]. The lens is particularly amenable to such analysis. Most other tissues are composed of multiple cell types and infiltrated by elements of the vascular or nervous systems. In such heterogeneous systems, determining the cellular origin of identified proteins can be problematic. Furthermore, in most cells, the plasma membrane represents only a small portion (<10%) of the total cellular membrane content and complicated fractionation protocols are required to isolate a plasma membrane-enriched sample. In the lens, intracellular organelles are degraded during the later stages of fiber cell differentiation [8]. As a result, organelles are restricted to a thin layer of fiber cells near the lens surface. In the majority of lens fiber cells, therefore, the plasma membrane is the only membrane system present.

In the current study, we used MudPIT analysis to further characterize the lens fiber cell membrane proteome. In addition to confirming the presence of several well-studied lens proteins, our analysis identified proteins not previously described in the lens and offered a semiquantitative determination of the relative expression levels of the various components.

## Methods

### Mice

Lenses from 121 18-28 day-old C57/BL6 mice sacrificed in the course of other studies were collected. Mice were killed by CO_2_ inhalation. Eyes were enucleated and the lenses were removed through an incision in the back of the eye. The lens capsule and adherent epithelium were dissected and discarded and the remaining fiber cell masses were stored at -80 ^°^C until use. All the procedures described herein were approved by the Washington University Animal Studies Committee.

### Preparation of lens membrane and soluble protein

Lenses were thawed and homogenized in 6 ml of 20 mM sodium phosphate buffer (pH 7.0) containing 1 mM EGTA, and the membrane pellet isolated by centrifugation at 20,000× g for 30 min. These centrifugation conditions were also used for the membrane pelleting and washing steps described below. The pellet was resuspended by brief vortexing and washed in 6.0 ml of homogenization buffer containing 50 mM dithiothreitol (DTT) and membrane proteins purified by a modification of the method of Russell et al. [9]. The pellet was resuspended in 0.6 ml of 7 M urea by probe sonication for 10 s at a setting of 3 using a model 60 Sonic Dismembrator (Fisher Scientific, Pittsburgh, PA), 0.6 ml of water were added, and the pellet isolated by centrifugation. The pellet was then suspended in 0.5 ml of ice cold 0.1 M NaOH containing 1 mM DTT, the suspension kept on ice for 15 min, and the pellet isolated by centrifugation. The pellet was then washed in 0.5 ml of 0.5 M Tris (pH 6.8) and suspended in 0.25 ml of homogenization buffer by brief sonication. This suspension of membrane proteins and the supernatant isolated following the initial centrifugation of the lens homogenate (soluble proteins) were then assayed for protein content using a BCA assay and bovine serum albumin standard (Pierce Chemical, Rockford, IL).

### Pepsinization and trypsinization of lens proteins

Eight-hundred μg of the recovered 1.2 mg of urea- and alkali-washed membrane fraction were used for a limited pepsin digestion of the membrane proteins using a modification of the method of Han and Schey [10]. The pellet was resuspended in 0.4 ml of 1.5 M Tris (pH 7.4): n-propanol (1:3 v/v) and reduction/alkylation of cysteine residues performed by addition of 10 µl of 0.9 M DTT, incubation at 37 °C for 15 min, addition of 10 µl of 1.0 M iodoacetamide (IAA), and incubation for 15 min at room temperature. An additional 10 µl of 0.9 M DTT were then added to assure all excess IAA was eliminated, the membrane pelleted, washed with 0.5 ml of water, and proteins delipidated by suspension in 0.5 ml of 95% ethanol at -20 °C overnight. The delipidated proteins were then pelleted and resuspended in 45.5 µl of 88% formic acid. The acid-solubilized membrane proteins were then diluted by ten stepwise additions of 35.5 µl of water to achieve a final acid concentration of 10%. Sixteen µl of freshly prepared 1 mg/ml porcine pepsin A (Worthington Biochemical, Lakewood, NJ) were then added (1:50 enzyme: substrate ratio), and the proteins incubated at 37 °C for 5 h in a shaking water bath to partially proteolyze the membrane proteins with the acid attenuated pepsin. Two mg portions of the soluble protein fraction were dried by vacuum centrifugation, dissolved in 8 M urea buffer, reduced/alkylated with DTT and IAA, diluted to a 2 M urea concentration, proteins trypsinized at 37 °C overnight at a ratio of enzyme/substrate of 1:25, and the reaction stopped by addition of formic acid as previously described [11]. The digests were then diluted by addition of 0.4 ml of water, and peptides isolated by solid phase extraction using a Sep-Pak Light cartridge (Waters, Milford, MA).

### MudPIT analysis of lens proteins

A combination of cation exchange and reverse phase chromatography steps were used to separate the complex mixture of lens membrane and soluble fraction peptides in preparation for tandem (MS/MS) mass spectrometric analysis. Solid phase extracted peptides from both the membrane and soluble protein fractions were separated into 39 and 44 fractions, respectively, using a 2.1×100 mm polysulfoethyl A column and KCl gradient, as described previously [11]. These fractions were then dried by vacuum centrifugation, dissolved in 100 µl of 5% formic acid, and 40 µl of each separated by reverse phase chromatography while collecting data-dependent MS/MS spectra on the eluted peptides. Peptides were separated using an Agilent 1100 series capillary LC system (Agilent Technologies Inc, Santa Clara, CA) and an LTQ linear ion trap mass spectrometer (ThermoFisher, San Jose, CA). Electrospray ionization was performed with an ion max source fitted with a 34 gauge metal needle and 2.4 kV potential. Samples were applied at 20 μl/min to a trap cartridge (Michrom BioResources, Inc, Auburn, CA), and then switched onto a 0.5×250 mm Zorbax SB-C18 column with 5 µm particles (Agilent Technologies) using a mobile phase containing 0.1% formic acid, 7-30% acetonitrile gradient over 95 min, and 10 μl/min flow rate. Data-dependent collection of MS/MS spectra used the dynamic exclusion feature of the instrument control software (repeat count equal to 1, exclusion list size of 50, exclusion duration of 30 s, and exclusion mass width of -1 to +4 ) to obtain MS/MS spectra of the three most abundant parent ions following each survey scan from m/z 400-2000. The tune file was configured with no averaging of microscans, a maximum inject time of 200 msec, and AGC targets of 3×10^4^ in MS mode and 1×10^4^ in Msn mode.

### Informatics

The analysis of the lens membrane and soluble protein digests produced 269,200 and 224,484 MS/MS spectra, respectively. These spectra were used to create DTA files using BioWorks 3.2 (ThermoFisher) with a molecular weight range of 550 to 4,000, an absolute threshold of 500, group scan setting of 1, a minimum of 25 ions, and a charge state analysis using the ZSA algorithm. A mouse species subset of the Sprot (v57.2) protein database (16,123 proteins) was prepared with concatenated reversed entries (and common contaminants) and searched with SEQUEST (ThermoFisher). Parent ion and fragment ion tolerances of 2.5 and 1.0 Da were used with calculated average and monoisotopic masses, respectively. Cysteine had a static modification mass of +57 Da, and no enzyme specificity was chosen. An in-house suite of programs [12] was used to provide a Peptide Prophet-like discriminant function [13] scoring to identify “correct” peptides and discard “incorrect” peptides using sequence-reversed matches to estimate false discovery rates. Of the 269,200 membrane protein spectra, 14,758 passed thresholds, with 162 matches to reversed sequences, giving an estimated peptide false discovery rate of 1.1%. In comparison, the analysis of the 224,484 soluble protein spectra resulted in 32,448 spectra passing thresholds, with 593 matches to reversed sequences, giving an estimated peptide false discovery rate of 1.9%. Protein identification lists were prepared using DTASelect v1.9 [14] with post processing of results to improve spectral count accuracy and allow strict protein identification criteria. Proteins were required to have two or more peptides with distinct sequences, and have a unique peptide count greater than or equal to one. Different charge states of the same peptide sequence were not considered as unique peptides. There were 350 non-redundant proteins identified in the membrane protein data set with two protein matches to reversed sequences. After removal of 16 common contaminants and 21 proteins having insufficient unique peptide evidence for confident identifications, a total of 313 non-redundant membrane proteins remained. A similar analysis of the soluble protein fraction resulted in the identification of 361 proteins with two matches to reversed sequences. Spectral counts of peptides shared between proteins were partitioned between the proteins on the basis of relative unique peptide evidence so that families of proteins having large numbers of shared peptides would have more accurate estimated total spectral counts.

The combined list of proteins found in both membrane and soluble protein fractions contained 575 non-redundant proteins. There were 197 proteins unique to the lens membrane fraction, 221 proteins unique to the lens soluble fraction, and 157 proteins in common. Of the 157 proteins in common, there were 35 that contained five or more spectral counts in the membrane fraction and showed a greater than twofold increase in spectral counts between the membrane prep sample and soluble lens sample and were thus classified as membrane enriched. Therefore, the total number of confident membrane protein identifications (unique plus enriched) was 232. Functional annotations were added to the protein results lists using the Protein Information and Property Explorer (PIPE) [15]. The list of all identified peptides and proteins in both membrane and soluble protein fractions, along with their spectral counts, designation as membrane unique or enriched, and annotations are found in Appendix 1.

### Semiquantitative molar expression ratio

A potential advantage of the MudPIT technique is that it provides a semiquantitative measure of relative protein abundance. There are several caveats, however. Clearly, a number of factors influence the spectral counts for a particular protein. For example, a protein would be underrepresented or even undetected if, by virtue of its tertiary structure or association with other elements, it were not efficiently cleaved during pepsin treatment. Similarly, certain peptides may be less stable than others and, therefore, be underrepresented in the final sample. One parameter that strongly effects the representation of a peptide in the final mix is the size of the parent protein. At equimolar concentrations, a large protein will generate more peptides than a small one of similar composition and structure. To normalize for this effect, we divided the spectral count for a given protein by the molecular mass of that protein so the abundance shown in the pie charts more closely resemble molar concentrations than weight concentrations. We recognize that the relationships may not be strictly linear and accordingly use the term “apparent abundance” when comparing the signals from two proteins. We note, however, that the lens membrane proteins shown here to have the greatest “apparent abundance” (MIP, Lim2, Gja3, Gja8, N-Cad, etc.) have also been shown in numerous published studies to be the most abundant proteins in the lens membrane.

### Lens preferred expression index (LPEI)

Lattin et al. [16] recently reported the results of a comparative microarray analysis in which gene expression was evaluated in a panel of 96 tissues and cell types, including the lens. We used these data to calculate a lens-preferred expression index (LPEI) for transcripts encoding each protein identified in the fiber cell membrane proteome. The LPEI was calculated for each probe set on the array by dividing the lens hybridization signal by the average for the other cell types. If a given gene was represented by multiple probe sets, the probe with the strongest lens signal was used to compute the LPEI. Genes expressed strongly or uniquely in the lens are expected to have a high LPEI. Mip, for example, has an LPEI of 1,261.

## Results and Discussion

The 232 proteins identified in the lens fiber cell membrane sample included Type I, Type II, Type III (multi-pass), lipid-anchored, and GPI-anchored membrane proteins, in addition to membrane-associated cytoskeletal elements and extracellular matrix components. In this analysis, the entire fiber cell mass was utilized. Thus, the sample also included a small proportion of cells originating from the surface layers of the lens. This superficial layer contains metabolically active cells that contain a full complement of cytoplasmic organelles. Ninety three proteins known or suspected to be components of intracellular organelles were detected in the membrane proteome. Presumably, these proteins originated in the organelle-rich, superficial fiber cell layer. A spreadsheet showing the identified peptides and proteins in the lens membrane and soluble protein fractions, spectral counts, percent coverage, designation as either membrane unique or enriched, functional annotations, and links to the UniProt database are included as a supplemental data file (Appendix 1).

**Figure 1:**
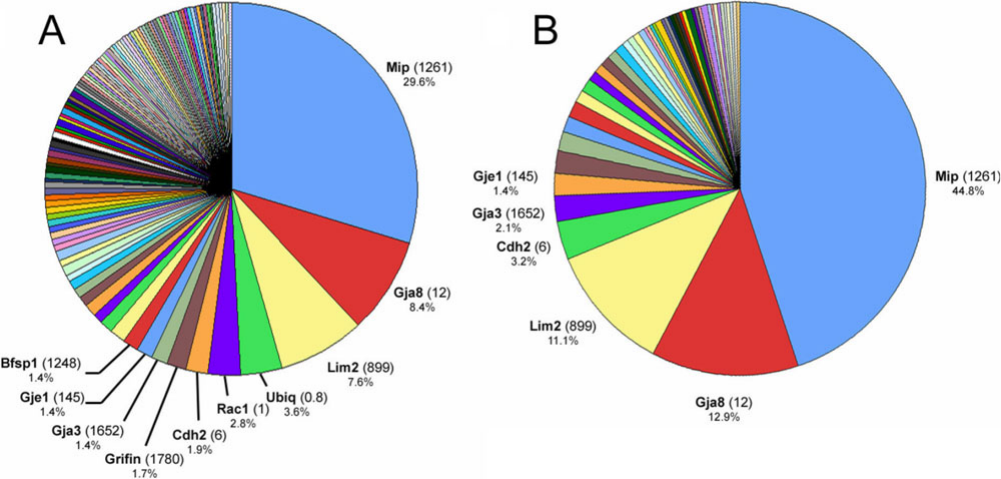
MudPIT analysis of the lens fiber cell membrane proteome. **A**: More than 200 membrane proteins and membrane-associated proteins were identified. The ten proteins of highest apparent abundance are indicated. For each protein, the number shown in parentheses is the lens preferred expression index (LPEI), a measure of the relative level of expression of the RNA for that protein in the lens compared to a panel of 96 other tissues and cell types (see text for details). The percentage value for each protein relates to its representation on that particular pie chart. **B**: Eighty-seven integral membrane proteins were detected. Six proteins (including three connexin proteins; Gja3, Gja8, Gje1) account for >75% of the integral plasma membrane proteins detected in MudPIT analysis of lens fiber membranes.

The protein with the highest apparent abundance in the lens fiber membrane (accounting for 29.6% of the total membrane protein [Figure 1A] and 44.8% of the integral membrane protein [Figure 1B]) was Mip, consistent with numerous biochemical studies that have shown this to be the most abundant of lens membrane proteins. Mip (a.k.a. Aqp0) is the founder member of the aquaporin family of water channels and is believed to be a bifunctional protein in the lens. Osmotic swelling measurements have confirmed that Mip functions as a water channel in vesicles prepared from native lens membranes [17] but structural studies have suggested that Mip may also function as a cell-cell adhesion protein [18]. Mutations in *MIP* in humans [19] and mice [20] result in cataracts. In addition to integral proteins such as Mip, cytoskeletal elements, including the lens specific intermediate filament protein Bfsp1, were also among the most abundant proteins identified in the membrane preparation (Figure 1A). An unexpectedly abundant component of the lens membrane proteome was ubiquitin. Ubiquitin is attached covalently to proteins prior to their degradation by the 26S proteasome. Long chains of ubiquitin monomers are assembled on substrate cytosolic proteins, whereas membrane proteins are usually monoubiquitinated. From the present data it is not possible to determine the identity of the ubiquitinated substrates.

Eighty-seven integral membrane proteins were localized to the lens fiber cell plasma membrane on the basis of GO component classification and/or previously published experimental studies (Figure 1B). Of these, six proteins (Mip, Gja8, Lim2, Cadh2, Gja3 and Gje1) accounted for more than 75% of the integral proteins detected.

To facilitate analysis and discussion, membrane proteins have been grouped into a number of functional categories: transporters, pumps, and channels; adhesion proteins; membrane cytoskeleton; cell signaling; Ras superfamily; and cell matrix interactions. A few proteins could not be accommodated readily in these categories and are listed under “miscellaneous” in Appendix 1.

### Transporters, pumps, and channels

**Figure 2:**
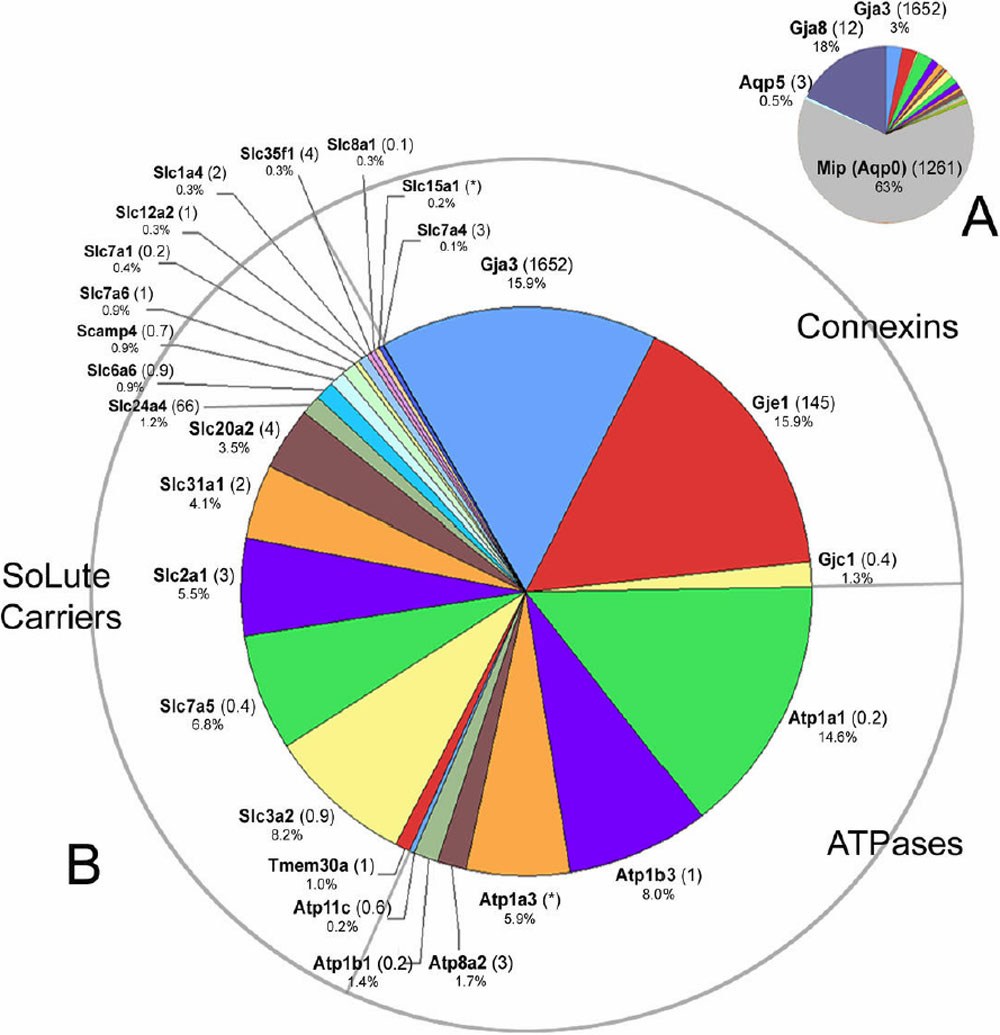
Transport proteins detected in the lens membrane proteome. **A**: Mip, a lens-specific aquaporin protein, and Gja8, a connexin protein, together dominate this category of proteins. **B**: To better visualize the results, Mip and Gja8 have been removed from the data set. Three classes of membrane transport protein were detected: connexins, ATPases and Slc transporters. The numbers in parentheses indicate the lens preferred expression index (LPEI, see text for details). Occasionally, it was not possible to calculate the LPEI because of inadequate hybridization signals, these instances are indicated by an asterisk.

In this category are grouped proteins believed to function in the active or passive movement of solute (or, in the case of aquaporins, water) across the cell membrane (Figure 2). Also included are the connexins, which facilitate the intercellular diffusion of ions and small metabolites.

Two aquaporins were detected in this analysis, Mip and Aqp5 (Figure 2A). Aqp5 has been identified previously in the bovine lens at the protein level [21] and Aqp5 transcripts have been detected in an EST analysis of human lens samples [22]. Aqp5 was present at modest levels (~100 fold lower than Mip) and it remains to be seen whether Aqp5 has an important physiological role. We note that no lens phenotype has been reported in Aqp5^-/-^ mice [23]. As shown in Figure 2A, Mip and Gja8 together account for 84% of the proteins in the *transporters, pumps, and channels* category. These proteins are so abundant that they mask the expression of other components. In Figure 2B, therefore, Mip, Aqp5 and Gja8 have been removed, to better visualize the expression of proteins of lesser abundance. In all, four connexins were identified in the lens fiber membrane proteome. In addition to Gja3 and Gja8, Gje1 and Gjc1 were detected. The avascular lens is known to rely on gap junction-mediated intercellular communication for ionic homeostasis [24]. It is expected, therefore, that gap junction proteins would be among the more prominent components of the membrane proteome. Gja8 and Gja3 have been studied intensively. In mice, gene knockout or chemical mutagenesis have demonstrated critical roles for Gja8 and Gja3 in lens growth and transparency [25]. Mutations in the human homologues of these genes have also been linked to cataract formation [26]. The LPEI values for Gja8 and Gja3 (12 and 1,652, respectively) indicate that these particular connexin proteins are among the most lens-specific of the membrane proteins. In comparison to Gja8 and Gja3, Gje1 is a much less well studied protein, although the current data suggest levels of expression for Gje1 that rival Gja3. Gje1 transcripts have been detected in the developing mouse lens and mutations in Gje1 cause cataracts and a variable small eye phenotype in mice [27]. Recently, Sonntag et al., [28] have shown that Gje1 (a.k.a. Gjf1, connexin23) protein forms hemichannels rather than patent gap junction channels in transfected HeLa cells. Although Gje1 is expressed in species ranging from zebra fish to cows, it appears to be present as a pseudogene in primates. The LPEI value for Gje1 (145) suggests that as with Gja3 and Gja8, Gje1 is a lens-specific membrane protein. Gjc1 (a.k.a. connexin 45) is approximately 10 fold less abundant that Gje1. Gjc1 is expressed in numerous tissues in the body, including the retina, as indicated by its low LPEI value of 0.4.

Na,K-ATPase is a ubiquitously expressed membrane protein that regulates the intracellular levels of Na and K. The Na,K-ATPase consists of two subunits: a large catalytic subunit (α) and a smaller glycoprotein subunit (β). In fiber cells, the α1 and β3 isoforms appear to be the most abundant, with lower levels of α3 and β1. Previous studies have detected α1 and α3 in the fiber cells [1], but there have been few previous reports on beta subunit expression. Despite its abundance in the fiber cell proteome, Na,K-ATPase enzyme activity is undetectable in mature fiber cells [29]. In other cells, Na,K-ATPase is often linked, via ankyrin, to the spectrin/actin cytoskeleton [30]. The lens, which is subject to mechanical stress during accommodation, has a well developed spectrin/actin cytoskeleton. It is possible, therefore, that the Na,K-ATPase retains its function as a membrane anchor point for the spectrin/actin lattice after its enzymatic role is over.

Two other plasma membrane ATPases, ATP11c and ATP8a2, were detected, albeit at low levels. These proteins are thought to function in phospholipid transport.

The solute carrier (Slc) designation includes a large group of membrane transport proteins with more than 300 members, organized into 47 families [31]. The lens membrane sample included 15 plasma membrane Slc members and four more Slc proteins thought to be localized to the inner mitochondrial membrane (Figure 2 and Table 1). Eight plasma membrane amino acid transporters were identified (Slc1a4, Slc2a1, Slc3a2, Slc6a6, Slc7a1, Slc7a4, Slc7a5, and Slc7a6). The two solute carriers with the greatest apparent abundance, Slc3a2 ( 4F2hc) and Slc7a5 (CD98), together comprise the large neutral amino acid transporter (Lat1), a heterodimeric membrane protein that preferentially transports neutral branched and aromatic amino acids. The Lat1 protein has also recently been shown to regulate the target of rapamycin complex 1 (mTorc1), a serine/threonine kinase with a pivotal role in activating cell growth [32]. Given the enormous increase in cell volume that accompanies fiber cell differentiation, the role of this pathway in the lens may merit further detailed investigation.

**Table 1:** Members of the solute carrier (Slc) family of transport proteins identified in the lens fiber cell membrane proteome.

**Gene symbol**	**Protein name (short name)**	**Substrate**
*Slc1a4*	Neutral amino acid transporter A	Glutamate/neutral amino acids
*Slc2a1*	Facilitated glucose transporter 2 (Glut-1)	glucose
*Slc3a2*	4F2 cell-surface antigen heavy chain (CD98) Forms heterodimeric transporter with *Lat1* or *Lat2*	Neutral amino acids (phenylalanine, tyrosine, leucine, and tryptophan)
*Slc6a6*	Sodium- and choloride-dependent taurine and beta-alanine transporters	Taurine, beta alanine
*Slc7a1*	High affinity cationic amino acid transporter 1 (Cat1)	Arginine, lysine and ornithine
*Slc7a4*	Cationic amino acid transporter 4 (Cat4)	Arginine, lysine and ornithine
*Slc7a5*	Large neutral amino acids transporter small subunit 1 (Lat1)	phenylalanine, tyrosine, leucine, arginine and tryptophan.
*Slc7a6*	Y+L amino acid transporter 2 (y+*Lat2*)	When co-expressed with Slc3a2 mediates the uptake of arginine, leucine and glutamine. Acts as an arginine/glutamine exchanger. Involved in sodium-independent uptake of dibasic amino acids and sodium-dependent uptake of neutral amino acids.
*Slc8a1*	Sodium/calcium exchanger 1 (*NCX1*)	Sodium, calcium
*Slc12a2*	Bumetanide-sensitive sodium – (potassium) chloride co-transporter 1 (Nkcc1)	Sodium, potassium, chloride
*Slc15a1*	Peptide transporter 1 (Pept1)	H+, oligopeptides
*Slc20a2*	Sodium-dependent phosphate transporter 2 (PiT-2)	phosphate
*Slc24a4*	Sodium/potassium/calcium exchanger 4 (Nckx4)	Sodium, potassium, calcium
*Slc31a1*	High affinity copper uptake protein 1 (Ctr1)	copper
*Slc35f1*	Solute carrier family 35 member F1	Putative nucleoside-sugar transporter
*Slc25a3	Phosphate transport protein (PTP)	phosphate
*Slc25a4	ADP/ATP translocase 1 (ANT1)	ADP,ATP
*Slc25a5	ADP/ATP translocase 2 (ANT2)	ADP, ATP
*Slc25a11	2-oxoglutarate/malate carrier protein (OGCP)	2-oxoglutarate, malate

The asterisk indicates that the protein is found in the inner mitochondrial membrane.

In addition to amino acid transporters, several important ion transport proteins were detected, including the bumetanide-sensitive Na^+^/K^+^/2Cl^-^ transporter (Nkcc1/Slc12a2), the Na^+^/Ca^2+^ exchanger (Ncx1/Slc8a1) and the Na^+^/K^+^/Ca^2+^ exchanger (Nckx4/Slc24a4). Nkcc1 has a well defined role in cellular volume regulation and both biochemical and physiological data support the expression of Nkcc1 in cortical lens fibers [33]. Membrane-based mechanisms that facilitate calcium extrusion are of particular interest in lens physiology, where calcium dysregulation is suspected to play a role in cataractogenesis. Calcium ions are transported actively by the plasma membrane Ca^2+^ ATPase (PMCA). However, no PMCA isoforms were detected in the plasma membrane proteome, consistent with earlier studies which noted epithelial rather than fiber expression of this protein [34]. Calcium may also be extruded from cells through a Na^+^/Ca^2+^ exchange (Ncx) mechanism or by K^+^-dependent Na^+^/Ca^2+^ exchange (Nckx). Significantly, the most abundant calcium extrusion protein detected in the current study was Slc24a4 (Nckx4). The expression levels of Slc24a4 RNA in the lens exceed that in any other tissue (LPEI = 66) yet, to our knowledge, the role of this protein in the regulation of lens fiber calcium has not been investigated. The ubiquitously expressed Slc8a1 (Ncx1) was also detected.

### Cell-cell adhesion proteins

**Figure 3:**
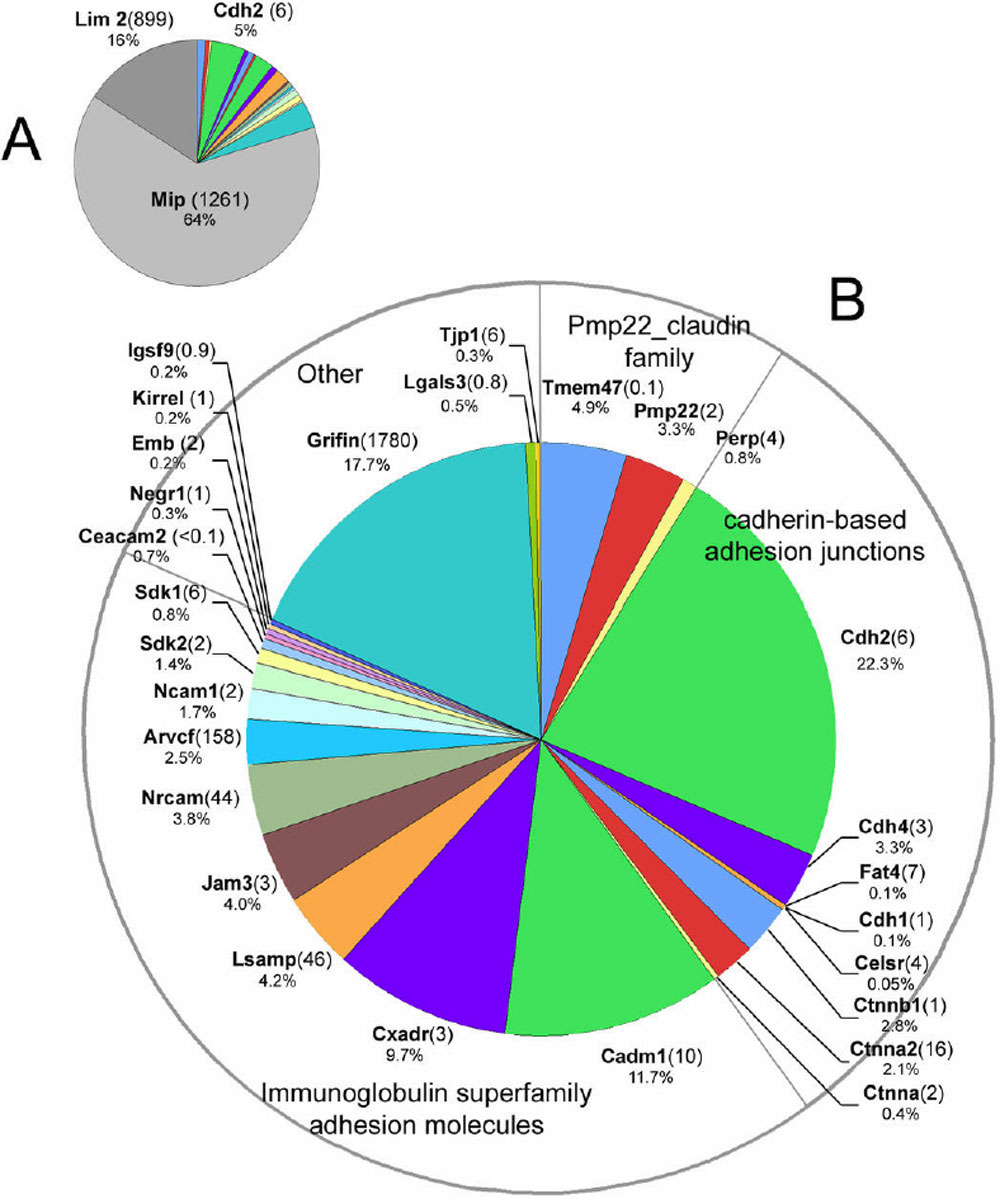
Cell adhesion proteins. **A**: Mip, Lim2, and Cdh2 were the adhesion proteins with highest apparent abundance (together constituting ≈ 85% of identified spectra). In **B**, Mip and Lim2 have been removed to help visualize the identities of adhesion proteins of lower apparent abundance. The numbers in parentheses indicate the lens preferred expression index (LPEI, see text for details).

As with the *Pumps, transporters and channels* grouping, the lens fiber *cell-cell adhesion protein* category is dominated by a few proteins expressed at very high levels. These are shown in Figure 3A and include Mip and Lim2. Lim2 (a.k.a. Mp20) is a member of the Pmp22_claudin protein family and may act as an adhesion molecule in the lens [35]. Disruption of the Lim2 locus in mice blocks the formation of fusions between lens cells and causes disturbances in the internal refractive properties of the lens [36]. The LPEI value for Lim2 is 899, indicative of a lens specific expression pattern. In Figure 3B, Mip and Lim2 have been removed from the data set to allow the relative abundance of the other proteins to be more clearly displayed. Lens cell-cell adhesion proteins belonged to three main groups: the Pmp22_claudin family, the cadherin family, and the immunoglobulin superfamily (IgSF).

Lim2, Tmem47, Pmp22 and Perp are members of the Pmp22_claudin family. Members of the family each contain four transmembrane segments. Claudins, the best studied members of the family, are components of epithelial tight junctions but were not detected in the fiber cell membrane proteome. Pmp22 (peripheral myelin protein 22) is a major component of myelin and mutations in Pmp22 are the cause of Charcot-Marie-Tooth disease Type1A [37]. Pmp22 plays an important role in myelination but its role in the lens is obscure. The expression of Tmem47 has not previously been described in the lens despite it being, after Lim2, the most abundant of the Pmp22_claudin members in the lens. In other cell types it is thought to be a component of maturing adherens junctions [38].

The existence of cadherin-based adherens junctions is supported by both morphological [39] and biochemical studies. In the current work, the predominant cadherin in the fiber cell membrane was Cdh2 (N-cadherin), first purified from lens tissue more than twenty years ago [40]. Conditional deletion of Cdh2 in the lens results in progressive disintegration of the fiber cells [41]. Traces of Cdh1 (E-cadherin), the predominant form in the lens epithelium [42], were also detected. Cdh4 (R-cadherin) expression was described first in the retina [43] but has been noted in the zebra fish lens [44]. Fat4 is a member of the evolutionarily ancient proto-cadherin family and was recently shown to regulate the planar cell polarity (PCP) pathway [45]. Fat4 has not been identified previously in the lens but accumulating evidence suggests that the PCP pathway has an important role in the determination of lens structure and ultrastructure [46,47]. The LPEI value of seven for Fat4 indicates that it is expressed at relatively high levels in the lens compared to most other tissues. At adherens junctions, cadherins are found complexed to catenin proteins that serve as links to the actin cytoskeleton and also have important signaling roles. Three catenins were identified in the fiber cell membrane sample. The catenin with the highest apparent abundance was beta 1 catenin (Ctnnb1), followed by alpha 2 and alpha 1 catenin. Arvcf (armadillo repeat gene deleted in velo-cardiofacial syndrome) is an IgSF member of the p120 (ctn) catenin subfamily known to associate with classical cadherins. Although not previously detected at the protein level, ARVCF transcripts have been identified in cDNA libraries prepared from human lenses [22]. The LPEI value for Arvcf is 168, suggesting unusually high expression levels of this gene in the lens compared to other tissues.

Members of the immunoglobulin superfamily (IgSF) are characterized by the presence of one or more extracellular immunoglobulin domains. The IgSF includes members that function as cytokine and growth factor receptors, antigen-presenting molecules and calcium-independent cell adhesion proteins. Eight IgSF members with demonstrated or suspected roles in intercellular adhesion were identified in the fiber cell membrane sample. The LPEI value for these 8 proteins ranged from 2-158 (mean=34) reflecting the importance of adhesive complexes in lens biology. The IgSF adhesive protein with the highest apparent abundance was Cadm1 (a.k.a. Tslc1, Syncam 1, Igsf4, Necl2). Cadm1 has not previously been described in the lens but is widely expressed in the central nervous system (including the retina), where it has an important role in driving synaptic assembly [48]. Cadm1 facilitates homophilic and heterophilic adhesive interactions. Loss of Cadm1 in mice causes male infertility [49] but no lens phenotype has been noted.

In addition to Cadm1, other adhesion proteins that are strongly expressed in the nervous system were identified in the lens membrane. For example, LSAMP (limbic system associated membrane protein), a protein that is enriched in the cortical and subcortical regions of the limbic system [50], was readily detected. Neuron-glia-CAM-related cell adhesion molecule (Nrcam) and Neural cell adhesion molecule 1 (Ncam1) have both been identified in lens membrane preparations previously. Mice deficient in Nrcam are viable and fertile but exhibit profound cataracts by six weeks of age, implying an essential role for Nrcam in the lens [51]. The apparent abundance of Ncam1 in the fiber cell membrane was approximately half that of Nrcam, consistent with previous studies suggesting that Ncam1 is primarily expressed in the lens epithelium with reduced levels of expression in the fiber cell compartment [52]. Sidekick (Sdk) proteins are synaptic adhesion molecules that play central roles in the targeting of neurites to appropriate sublaminae in the retina [53]. They have not been identified previously in the lens and their role there is unknown.

Two related molecules, junction adhesion molecule 3 (Jam3) and the coxsackie and adenovirus receptor (Cxadr), with well known roles in tight junction formation and stability [54,55], were also identified. Tight junctions are not believed to exist in the lens fiber cell compartment so presumably Jam3 and Cxadr have other functions in the lens. Both molecules are known to interact with ZO1 (Tjp1) through their PDZ-binding domains. ZO1 (Tjp1) was detected in the fiber cell membrane proteome, where it has previously been shown to interact with Gja3 and Gja8, the two major fiber cell connexins [56].

A prominent component of the membrane proteome was Grifin (galectin-related interfiber protein). It is included here with the grouping of adhesive proteins solely on the basis of its expression pattern (immunofluorescence analysis suggests that Grifin accumulates at the interstices between lens fiber cells [57]) for there are no direct experimental data regarding the function of this protein. Grifin is expressed uniquely in the lens (LPEI = 1780), at levels more normally associated with crystallin proteins [57]. Unlike true galectins, however, Grifin lacks the ability to bind beta-galactoside sugars and its role is currently a mystery.

### Membrane cytoskeleton

**Figure 4:**
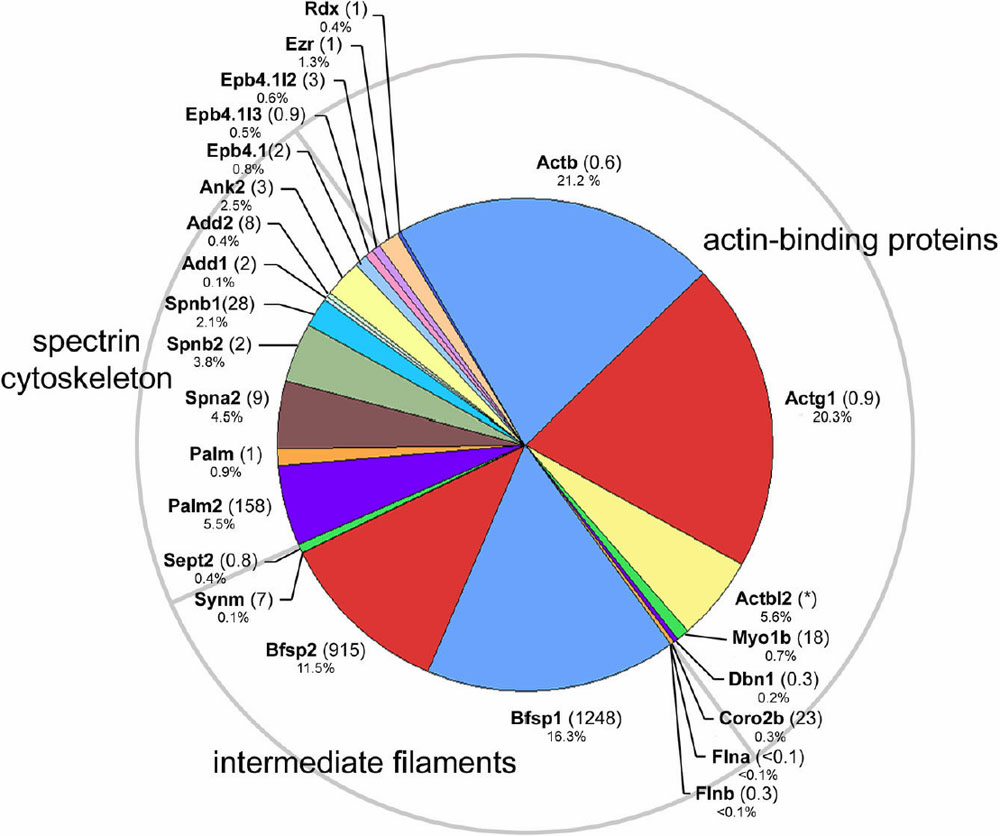
Components of the membrane cytoskeleton identified in the fiber cell membrane proteome. Actin-binding proteins, intermediate filament proteins and elements of the spectrin cytoskeleton were detected. The numbers in parentheses indicate the lens preferred expression index (LPEI, see text for details). Occasionally, it was not possible to calculate the LPEI because of inadequate hybridization signals, these instances are indicated by an asterisk.

Several cytoskeletal, membrane-associated proteins were identified in the fiber cell membrane proteome (Figure 4). Unsurprisingly, two widely expressed cytoplasmic non-muscle actins (beta actin [Actb] and gamma1 actin [Actg1]) were detected, in addition to actin beta-like 2 (Actbl2) and several actin-binding proteins (including filamin A and B [Fina and Finb], drebrin 1 [Dbn1], myosin 1b [Myo1b], and coronin 2b [Coro2b]).

Bfsp1 and Bfsp2 (a.k.a. filensin and CP49, respectively), two abundant, lens-specific (LPEI values of 1248 and 915, respectively) intermediate filament proteins, co-purified with the lens membranes. Filensin and CP49 are thought to be tethered to the fiber cell membrane in part via an interaction with the C terminus of Mip [58]. Synemin, an intermediate filament protein enriched at the sarcomere of skeletal myocytes was detected in the lens fiber membrane proteome, albeit it at much lower levels than either filensin or CP49. Sept2, a member of the septin family of cytoplasmic filaments was also detected. Septins have essential functions in cytokinesis but their role in the strictly post-mitotic fiber cells is obscure.

Palm 1 (paralemmin 1) and 2 are prenylated, palmitoylated proteins that associate with the cytoplasmic face of plasma membranes and are thought to be implicated in membrane dynamics and regulation of cell shape. Palm 1 is a Pax6-responsive gene and its occurrence in the lens has been noted previously [59,60]. The present data, however, suggest that Palm2 may be the more abundant and lens specific (LPEI=158) of the two lens fiber paralemmins.

Several components of the spectrin/actin sub-membrane cytoskeleton were identified, including alpha 2 spectrin (Spna2), beta 1 spectrin (Spnb1), beta 2 spectrin (Spnb2), and ankyrin 2 (Ank2; aka ankyrin B). In the red cell membrane, ankyrin links the spectrin lattice to integral membrane proteins such as band 3. In the lens, ankyrin is essential for tissue transparency [51]. The fiber cell membrane proteome also contained several band 4.1 proteins (Epb4.1, Epb4.1-like 2, Epb4.1-like3), and adducin 1 and 2 (Add1 and 2). In red cells, these components form a ternary complex which defines the nodal junctions of the membrane-skeletal network and through attachment to integral membrane proteins connects the spectrin/actin meshwork to the membrane [61]. The band 4.1 superfamily includes the ERM (ezrin, radixin, moesin) proteins, which link actin (directly or indirectly) to integral membrane proteins. Two ERM proteins, ezrin and radixin were identified in the present study, confirming an earlier observation by Maisel and colleagues [62].

### Cell signaling

**Figure 5:**
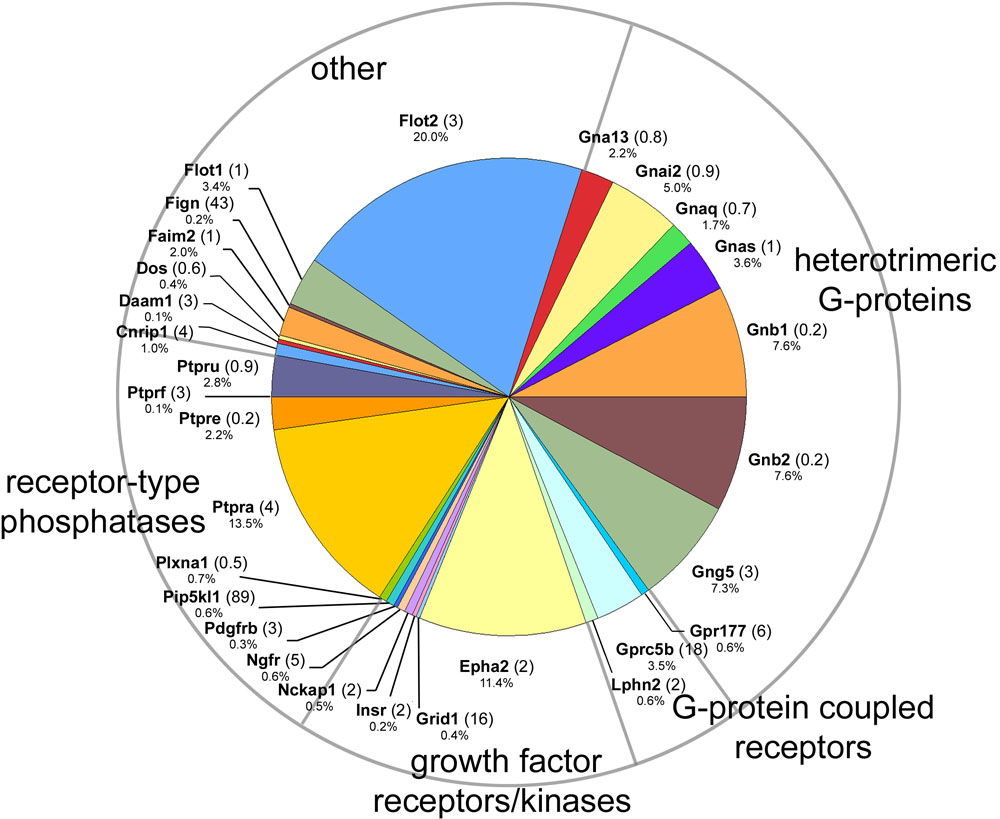
Cell signaling components identified in the fiber cell membrane proteome.

Several classes of signaling molecules were identified in the lens fiber cell membrane proteome (Figure 5).

Heterotrimeric guanine nucleotide-binding proteins (G-proteins) are ubiquitously-expressed receptor/effector coupling molecules. Alpha (Gna13, Gnai2, Gnaq), beta (Gnb1, Gnb2), and gamma (Gng5) G-protein subunits were detected in the lens membrane proteome. Three G-protein-coupled receptors were identified, Latrophilin 2, Gpr177 and Gprc5b. Gpr177 was recently shown to encode Wls/Evi, a protein that is essential for the secretion of Wnt proteins. Wnt signaling plays a critical role in patterning of embryos and control of cyto-architecture in several tissues, including lens [47]. Gprc5b (aka Raig2) is an orphan, retinoic acid-inducible G-protein-coupled receptor. Microarray analysis indicates that this gene is expressed at higher levels in the lens than other tissues (LPEI=18).

Several protein tyrosine kinase growth factor receptors were detected in the fiber cell membrane proteome. The receptor with highest apparent abundance was Epha2. Interestingly, potential ligands for Epha2, for example ephrin A1, were not detected in the current study. Epha2 has been implicated in both inherited and age-related cataract [3,63]. Other growth factor receptors included insulin receptor (Insr), nerve growth factor receptor (a.k.a. p75, neurotrophic receptor) and platelet derived growth factor receptor B (PDGFRB). PlexinA1 (Plxna1) is part of a multimeric receptor complex. Semaphorin 3A (a ligand for Plxna1) is secreted by the lens epithelium [64]. Semaphorins are secreted axonal guidance molecules generally functioning to deflect axons from inappropriate targets. Lens epithelium-derived semaphorin 3A regulates innervation of the embryonic cornea but whether it also signals via plexinA1 receptors located in the fiber cell membrane, and to what effect, has not yet been tested.

Four different receptor-type protein tyrosine phosphatases (Ptpru, Ptprf, Ptpre, and Ptpra) were detected. The phosphatase with highest apparent abundance was Ptpra, an enzyme which dephosphorylates and activates Src family kinases [65] and, thus, influences several important cellular processes, including cell cycle kinetics, integrin signaling, and cell-cell adhesion.

In the “Other” category are grouped signaling molecules that do not readily fall into one of the other designations. Flotillins (Flot1 and Flot2) are concentrated at caveolae, small indentations in the plasma membrane enriched in signaling molecules. Although they are relatively abundant proteins in the lens fiber cell membrane, the precise function of flotillins has yet to be determined. Fidgetin (Fign) is a AAA+ ATPase reported previously in the mouse lens [66]. The fidget mouse, resulting from a mutation in the fidgetin gene, exhibits cataracts and microphthalmia in addition to extraocular defects.

### Ras superfamily of small GTPases

**Figure 6:**
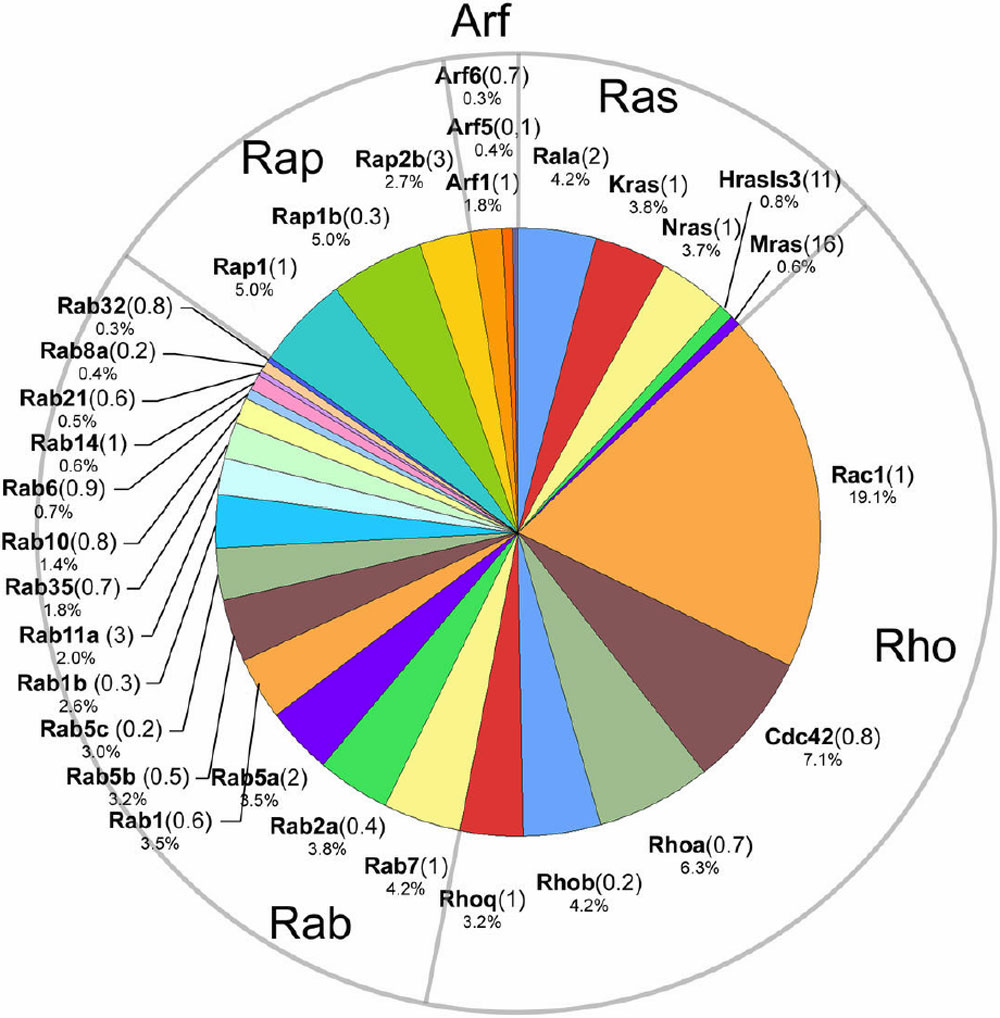
Members of the Ras superfamily of small GTPases identified in the fiber cell membrane proteome.

The Ras superfamily of small GTPases was well represented in the fiber cell membrane proteome (Figure 6) although, for most family members, the LPEI values were unremarkable, indicating that expression levels in the lens were similar to other tissues. Members of the Ras, Rho, Rab, Rap, and Arf families were detected. Many of the small GTPases function in membrane trafficking and are located in the Golgi or endosome compartments. Presumably, therefore, most of these proteins originated in cells located near the lens surface, for only the superficial layer of lens cells contain the full complement of cytoplasmic organelles.

Rho proteins are ubiquitously expressed molecules that play a pivotal role in actin polymerization and stress fiber formation. Rho, Rac, and Cdc42 have previously been identified in the lens. Disruption of Rho family GTPase activity by transgenic expression of Rho GDP dissociation inhibitor alpha (GDIα) results in abnormalities in the migration pattern, elongation and organization of lens fiber cells [67]. During lens fiber cell elongation the surface area of the cells increases at a rate of 6000 μm^2^/day [68]. However, there have been few studies on lens membrane trafficking. Rab and Arf proteins play critical roles as regulators of vesicular transport and membrane trafficking. Rab7 and Rab5b, two of the more abundant Rab family proteins identified in the lens membrane proteome, have been detected in lens endosomes previously, where they co-localize with components of the TGFβ signaling pathway [69].

### Cell matrix interaction

**Figure 7:**
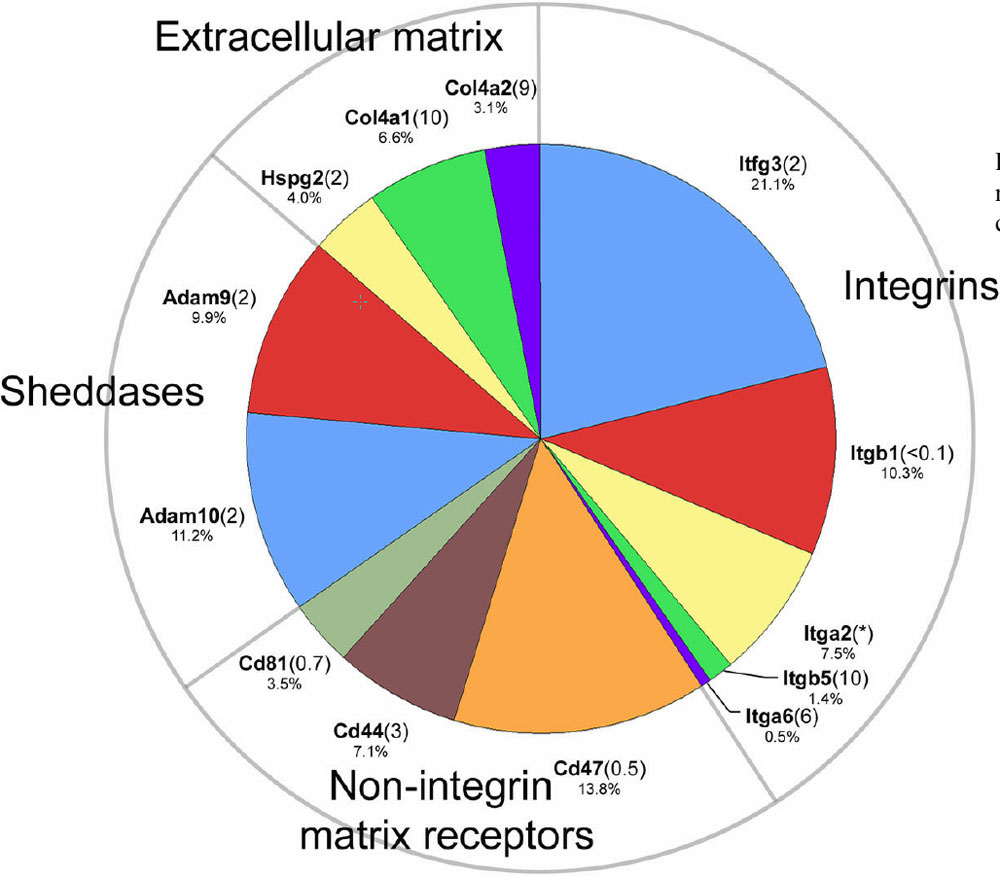
Extracellular matrix and matrix-interacting proteins in the fiber cell membrane proteome.

In preparing lens membrane samples for analysis, we removed by dissection the collagenous lens capsule and adhering lens epithelial cells. Nevertheless, extracellular matrix components and their receptors were still detected in the membrane proteome (Figure 7). Type IV collagen and perlecan (Hspg2) were identified. Perlecan is essential for lens capsule integrity and lens transparency [70]. It is possible that collagen and perlecan were derived from pieces of capsule that remained attached to the fiber cell mass after dissection. Alternatively, these proteins may have originated in the endoplasmic reticulum of the nucleated outer fiber cells.

Most of the integrin subunits identified in the fiber proteome have been previously documented in the lens [71], where they are known to play critical roles. Beta1 integrin (Itgb1), for example, is a component of the basal membrane complex, where it mediates the attachment of the posterior fiber tips to the lens capsule [72]. Lens specific deletion of beta1 integrin results in cell death in the lens epithelium and disintegration of lens fibers [73]. Itfg3 (integrin alpha GF-GAP repeat containing 3) was the integrin with highest apparent abundance in the lens membrane sample. The presence of Itfg3 has not previously been reported in the lens. Although transcripts for this protein are detected in many tissues (LPEI=2) there is currently little information regarding its function. Three other, non-integrin, proteins believed to interact with the extracellular matrix were also identified, CD44, CD47, and CD81. CD44 is a cell surface glycoprotein that serves as a receptor for hyaluronan and has been implicated in a wide variety of cellular functions, including cell adhesion and migration. CD44 was recently described in the lens [74]. Mice lacking the *CD44* gene show no overt lens phenotype [74]. CD47 associates with and modulates the activity of several families of integrins. CD81 is a member of the tetraspanin family and like CD47 is also believed to complex with integrins. CD81 has a suspected role in myoblast fusion [75] but whether it plays an analogous role in lens cell fusion remains to be determined.

Finally, two “sheddases”, Adam9 and Adam10, were identified in the membrane proteome. Sheddases are membrane-bound enzymes that cleave the extracellular portions of receptors and other transmembrane proteins often to release bioactive, soluble ectodomains. The role of Adams in lens cell biology in unknown, although downregulation of Adam9 accompanies the development of anterior polar cataracts [76].

### Summary

This analysis represents an effort to catalog systematically the expression profile of membrane proteins in mouse lens fiber cells. Previous studies in other species have utilized laser microcapture [21] or MALDI tissue imaging techniques [77] to examine the composition of the lens membranes as a function of location within the tissue. In conjunction with microarray and EST-based analysis of the fiber cell transcriptome [22,78] the current dataset should be useful for gene discovery purposes or to identify candidate genes in genetic screens. One advantage of the MudPIT approach is that it provides a (reasonably unbiased) semi-quantitative measure of the relative abundance of the various components. Thus, for the first time, it is possible to construct a tentative model of the lens fiber membrane and discriminate between major and minor components. The current data were obtained from young (<one month old) mice. It will be of interest to perform similar studies on the lenses of aged animals. Due to a unique growth pattern any lens contains cells of all ages and stages of differentiation. In future studies it may be possible to subdivide the fiber cell mass and thereby examine age-dependent changes in membrane composition. Such experiments will, however, require more sensitive instrumentation than used here to permit the analysis of smaller tissue volumes. In recent years investigators have generated mice deficient in some of the most abundant fiber cell membrane proteins (e.g. Mip, Gja3, Gja8, Lim2). A comparative analysis of the lenses from such mice may reveal how the absence of such major components impacts the fiber cell membrane proteome as a whole. Finally, it would be of great interest to compare the present data with a similar analysis of the human lens. Such an analysis might help identify a characteristic core set of membrane proteins that play key roles in all lenses.

## Appendix 1

Mouse fiber cell membrane and soluble protein peptide identification summary. To access the data, click or select the words “Appendix 1.” This will initiate the download of a Microsoft Excel (.xls) file file.
